# *Lactobacillus amylovorus* Inhibits the TLR4 Inflammatory Signaling Triggered by Enterotoxigenic *Escherichia coli* via Modulation of the Negative Regulators and Involvement of TLR2 in Intestinal Caco-2 Cells and Pig Explants

**DOI:** 10.1371/journal.pone.0094891

**Published:** 2014-04-14

**Authors:** Alberto Finamore, Marianna Roselli, Ambra Imbinto, Julie Seeboth, Isabelle P. Oswald, Elena Mengheri

**Affiliations:** 1 Consiglio per la Ricerca e la Sperimentazione in Agricoltura (CRA), Centro di Ricerca per gli Alimenti e la Nutrizione (Research Center on Food and Nutrition, CRA-NUT), Rome, Italy; 2 INRA, UMR 1331 Toxalim, Research Center in Food Toxicology, Toulouse, France; 3 University of Toulouse, National Polytechnic Institute of Toulouse (INP), UMR 1331 Toxalim, Toulouse, France; Charité, Campus Benjamin Franklin, Germany

## Abstract

Inflammation derived from pathogen infection involves the activation of toll-like receptor (TLR) signaling. Despite the established immunomodulatory activities of probiotics, studies relating the ability of such bacteria to inhibit the TLR signaling pathways are limited or controversial. In a previous study we showed that *Lactobacillus amylovorus* DSM 16698^T^, a novel lactobacillus isolated from unweaned pigs, protects the intestinal cells from enterotoxigenic *Escherichia coli* (ETEC) K88 infection through cytokine regulation. In the present study we investigated whether the ability of *L. amylovorus* to counteract the inflammatory status triggered by ETEC in intestine is elicited through inhibition of the TLR4 signaling pathway. We used the human intestinal Caco-2/TC7 cells and intestinal explants isolated from 5 week-old crossbreed Pietrain/Duroc/Large-White piglets, treated with ETEC, *L. amylovorus* or *L. amylovorus* cell free supernatant, either alone or simultaneously with ETEC. Western blot analysis showed that *L. amylovorus* and its cell free supernatant suppress the activation of the different steps of TLR4 signaling in Caco-2/TC7 cells and pig explants, by inhibiting the ETEC induced increase in the level of TLR4 and MyD88, the phosphorylation of the IKKα, IKKβ, IκBα and NF-κB subunit p65, as well as the over-production of inflammatory cytokines IL-8 and IL-1β. The immunofluorescence analysis confirms the lack of phospho-p65 translocation into the nucleus. These anti-inflammatory effects are achieved through modulation of the negative regulators Tollip and IRAK-M. We also found that *L. amylovorus* blocks the up-regulation of the extracellular heat shock protein (Hsp)72 and Hsp90, that are critical for TLR4 function. By using anti-TLR2 antibody, we demonstrate that TLR2 is required for the suppression of TLR4 signaling activation. These results may contribute to develop therapeutic interventions using *L. amylovorus* in intestinal disorders of piglets and humans.

## Introduction

The intestinal mucosa is colonized by a vast community of bacteria and should be able to defend against pathogen infections. The Toll-like receptor (TLR) family plays a critical role in the host defense or in the development of inflammation by recognizing microbe-associated molecular patterns. Among these receptors, TLR4 has been associated with pathogenesis of several diseases [1]–[4]. Indeed, binding of lipopolysaccharide (LPS) to TLR4 caused intestinal inflammation through production of pro-inflammatory cytokines [5], [6], and elimination of TLR4 increased the susceptibility to dextran sodium sulfate-induced disease [7]. In addition, the expression of TLR4 was increased in intestinal epithelial cells and dendritic cells of patients suffering of ulcerative colitis and Crohn's disease and in macrophages of inflamed tissues [8]–[10], while mice knockout for TLR4 showed reduced myocardial ischemic injury [11]. TLR4 was found to be the most strongly expressed TLR in porcine intestinal cells derived from neonatal pigs [6], that can be related to the high incidence of inflammation associated with pig weaning. TLR4 detects Gram-negative bacteria, but recent studies identified other molecules able to bind to and activate this receptor, namely the extracellular heat shock proteins (Hsps), such as the extracellular Hsp72 and Hsp90 [12]–[14]. When released from cells, these Hsps may induce inflammation in a TLR4- and NF-κB-dependent mechanism [15], [16], and circulating Hsp72 has been found increased in pathological conditions including renal disease, hypertension, atherosclerosis and sickle cell disease [17].

Induction of TLR4 may lead to inflammatory cytokine over-production through activation of two signaling pathways, the early myeloid differentiation primary response gene 88 (MyD88)-dependent and delayed MyD88-independent response [18]. The MyD88-dependent cascade includes activation of the NF-κB pathway, involving recruitment of the IL-1R-associated kinases (IRAKs), phosphorylation of IκB kinase (IKK) and subsequent phosphorylation and degradation of the family of IκB proteins, which allow phosphorylation of NF-κB followed by its translocation into the nucleus and transcription of pro-inflammatory cytokines such as TNF-α, IL-1β, IL-6 and IL-8 [19]–[22].

Targeting the TLR4-mediated inflammatory signaling may represent a way to counteract the pathogen induced damages. Probiotic bacteria are microorganisms that may confer health benefits to the host, including prevention of inflammatory intestinal diseases [23]–[25]. There is some evidence that probiotic bacteria can inhibit the activation of TLR4 signaling pathway, although the studies are limited and the results sometimes contradictory. For instance, a down-regulation of TLR4 expression by *L. paracasei* associated with a decreased cytokine and chemokine release against *Salmonella typhi* infection was found in dendritic cells [26]. *L. jensenii* reduced the mRNA level of pro-inflammatory cytokines by inhibiting the pathogen induced TLR4 activation in porcine intestinal epithelial cells [27]. However, it was also shown that *L. rhamnosus* and *L. plantarum* did not change the TLR4 expression neither the secretion of IL-8 in cells infected with *Salmonella* [28].

In a previous study, we showed that treatment of porcine intestinal cells with *L. amylovorus* strain 16698^T^ (formerly called *L. sobrius*), a new lactobacillus species isolated from intestine of unweaned piglets, protects against enterotoxigenic *E. coli* (ETEC) K88 infection by inhibiting pathogen adhesion and membrane damages through cytokine modulation [29]. This lactobacillus is able to reduce the diarrhea caused by ETEC, decrease colonization of ETEC, and improve weight gain of infected piglets [30]. Other than in piglets, the probiotic characteristics of *L. amylovorus* were shown in an *in vitro* model that simulates the human upper gastrointestinal tract, suggesting a potential use of *L. amylovorus* for animal as well as for human health [31]. In the present study we aimed to examine the ability of *L. amylovorus* to counteract the inflammatory stimulus triggered by ETEC in intestine through inhibition of the TLR4 signaling pathways and modulation of the negative regulators. We used an *in vitro* model of intestinal cells and *ex vivo* model of piglet explants that more closely mimics the gut mucosal environment [32]. We found in both human Caco-2/TC7 cells and pig intestinal explants, that *L. amylovorus* and its cell free supernatant are able to inhibit the different steps of TLR4 signaling activated by ETEC K88 and the production of inflammatory cytokines through modulation of the negative regulators Toll-interacting protein (Tollip) and IRAK-M, as well as down-regulation of the extracellular Hsp72 and Hsp90. We also show that TLR2 activation is required for these anti-inflammatory activities.

## Materials and Methods

### Ethic statement

All animal experiments were carried out in strict accordance with the recommendation of the European Guidelines for the Care and Use of Animals for Research Purposes. The protocol was approved by the Committee of the Ethics of Animal Experiments of Pharmacology-Toxicology of France Midi-Pyrénées, Tocométhique, agreement number TOXCOM/0017. This Committee is affiliated to INP and INRA. The two authors, Isabelle Oswald and Julie Sebooth are from INRA. All efforts were made to minimize suffering and immediately after electrical stunting, animals were killed by exanguination prior to samples collection, as already described [33].

### Epithelial cell culture

The human intestinal Caco-2/TC7 cell line (developed by Chantret et al. [34] and kindly provided by Monique Rousset, Institut National de la Santé et de la Recherche Médicale, INSERM, France) was used. These cells derive from parental Caco-2 cells at late passage, and have been reported to express higher metabolic and transport activities than the original line [35]. Cells were used between passages 100 and 105 and were routinely grown on plastic tissue culture flasks (75 cm^2^ growth area, Becton Dickinson, Milan, Italy) in Dulbecco's modified minimum essential medium (DMEM; 3.7 g/L NaHCO_3_, 4 mM glutamine, 10% heat inactivated fetal calf serum, 1% non essential amino acids, 10^5^ U/L penicillin and 100 mg/L streptomycin). All cell culture reagents were from Euroclone (Milan). The cells were routinely maintained at 37°C in an atmosphere of 5% CO_2_/95% air at 90% relative humidity. For the experiments, cells were seeded on Transwell filters (polyethylene terephtalate filter inserts for cell culture; Becton Dickinson) of 12 mm diameter, 0.45 µm pore diameter, as described below. After confluency, cells were left for 17–21 days to allow differentiation. Medium was changed three times a week.

### Pig jejunal explants

Jejunal tissues were obtained from five Pietrain/Duroc/Large-White piglets that were 5 week-old and weaned at 4 weeks (8.3±0.15 Kg), housed in groups in normal conditions, and fed a standard commercial diet (Solignac, Bessière, France), as previously described [36]. Briefly, the external *tunica muscularis* was removed from middle jejunum segment, then explants were excised with punch trocards (Centravet, Lapalisse, France) and placed in Williams culture medium (Sigma-Aldrich, St Louis, MO) supplemented with 1% penicillin/streptomycin, 0.5% gentamycin (Eurobio, Les Ulis, France), 4.5 g/L glucose (Sigma-Aldrich), 10% fetal bovine serum (Eurobio) and 30 mM amino acids (Ala/Glu, Eurobio).

### Bacterial growth

ETEC strain K88 (kindly provided by The Lombardy and Emilia Romagna Experimental Zootechnic Institute, Reggio Emilia, Italy) was grown in Luria-Bertani (LB) broth containing 1% tryptone and 0.5% yeast extract (both from OXOID, Basingstoke, England), plus 1% NaCl, pH 7.0. After overnight incubation at 37°C with vigorous shaking, bacteria were diluted 1∶200 in fresh LB and grown until mid-log phase. Bacterial cells were then harvested by centrifugation at 3000×g for 10 min at 4°C and resuspended in antibiotic- and serum-free DMEM.

*L. amylovorus* strain DSM 16698^T^_,_ isolated from piglet small intestine [37], was grown in DeMan Rogosa Sharp (MRS) medium (DIFCO, Milan) at 37°C under anaerobic conditions. After overnight incubation, bacteria were diluted 1∶15 in fresh MRS, grown until mid-log phase and processed as described above for ETEC. Bacterial concentrations were determined in preliminary experiments by densitometry and confirmed by serial dilutions followed by CFU counts of ETEC on LB agar after 16 hrs incubation, and of *L. amylovorus* on MRS agar after 48 hrs incubation at 37°C, under anaerobic conditions.

The viability of ETEC and *L. amylovorus* grown on DMEM did not differ from that of bacteria grown on LB or MRS media, as tested in preliminary experiments by CFU counts after agar plating of bacterial inocula from the different media.

### Preparation of *L. amylovorus* cell free culture supernatant

Cell free culture supernatant was prepared from overnight cultures of *L. amylovorus* after centrifugation at 4000×g for 10 min at 4°C followed by filtration of the supernatant fractions through a 0.22 µm pore-size filter, to remove any remaining bacteria. The cell free supernatant equivalent to 5×10^7^ CFU/mL was added to 1 mL antibiotic- and serum-free DMEM for the experiments described below. Since the addition of bacterial medium to DMEM can reduce the pH and previous studies reported that a pH value below 5.8 affects Caco-2 cell viability [38], the pH value of supernatants in DMEM was checked and resulted not lower than 6.

### Analysis of TLR4 signaling in Caco-2/TC7 cells and pig jejunal explants by Western blot

Caco-2/TC7 cells differentiated on Transwell filters (1×10^6^ cells/filter), were untreated (control) or apically treated with 1 mL of medium containing ETEC (5×10^6^ CFU/mL), *L. amylovorus* (5×10^7^ CFU/mL) or *L. amylovorus* supernatant (equivalent to 5×10^7^ CFU/mL), either alone or simultaneously with ETEC, for 2.5 hrs. We chose the pathogen concentration and time of incubation based on preliminary experiments to allow triggering of the inflammation pathway without disruption of the cell monolayer. The 1∶10 ratio of ETEC to *L. amylovorus* was that used in our previous study [29]. In some experiments, neutralizing anti-TLR2 antibody was apically added to Caco-2/TC7 cells in antibiotic- and serum-free DMEM (150 µg/L; R&D System Inc., Minneapolis, MN), for 1 hr before the addition of bacteria as above described.

Pig jejunal explants (6 mm diameters) were untreated (control) or treated with 2.5 mL of medium containing ETEC (5×10^6^ CFU/mL) or *L. amylovorus* (1.25×10^8^ CFU/mL), either alone or simultaneously, for 1.5 hrs at 39°C in a humidified atmosphere of 5% CO_2_. After treatment, tissues were collected, immediately snap-frozen and maintained at −80°C before analysis.

For analysis of the TLR4 cascade proteins, Caco-2/TC7 cells and intestinal explants were lysed or homogenized respectively, in cold radioimmunoprotein assay (RIPA: 20 mM Tris-HCl pH 7.5, 150 mM NaCl, 0.1% SDS, 1% Na deoxycholate, 1% Triton X-100) buffer supplemented with 1 mM phenylmethylsulphonyl fluoride, protease inhibitor cocktail (Complete Mini, Roche, Milan) and phosphatase inhibitor cocktail (PhosSTOP, Roche). For analysis of the extracellular Hsp72 and Hsp90, 30 µL of medium collected from basolateral compartment were added to 30 µL of 2× RIPA. The cells, explants and basolateral media were centrifuged at 15000×g for 20 min and supernatants were recovered. Cell lysates (50 µg total proteins) intestinal homogenates (50 µg total proteins) and 50 µL of basolateral media were dissolved in sample buffer (50 mM Tris-HCl, pH 6.8, 2% SDS, 10% glycerol, 100 g/L bromophenol blue, 10 mM β-mercaptoethanol), heated for 5 min, fractionated by SDS-polyacrylamide gel (4–20% gradient) electrophoresis and transferred to 0.2 µm nitrocellulose filters (Trans-Blot Turbo, Biorad, Milan). Membranes were incubated with the following primary antibodies: rabbit polyclonal anti-human TLR4, MyD88, IRAK-4, IKKα, IKKβ, phospho(P)-IKKα/β, IκBα, P-IκBα, NF-κB p65, P-p65, IRAK-M, Tollip, or mouse monoclonal anti-human Hsp90, Hsp72, α-tubulin antibodies. All primary antibodies were from Cell Signaling Technology (Danvers, MA), except for TLR4, that was from Santa Cruz Biotechnology Inc. (Santa Cruz, CA). Preliminary experiments verified the complete cross reactivity of the human antibodies with the analyzed pig proteins (data not shown). Proteins were detected with horseradish peroxidase-conjugated secondary antibodies (Cell Signaling Technology) and enhanced chemiluminescence reagent (ECL kit LiteAblot Extend, Euroclone), followed by analysis of chemiluminescence with the charge-coupled device camera detection system Las4000 Image Quant (GE Health Care Europe GmbH, Milan).

Relative levels of TLR4, MyD88, IRAK-4, Tollip and IRAK-M were normalized to α-tubulin, whereas the phosphorylated proteins were normalized to their corresponding unphosphorylated forms.

### TLR4 and P-p65 immunolocalization in intestinal cells

Caco-2/TC7 cells differentiated on Transwell filters (1×10^6^ cells/filter), were untreated (control) or apically treated with 1 mL of medium containing ETEC (5×10^6^ CFU/mL) or *L. amylovorus* (5×10^7^ CFU/mL), either alone or simultaneously, for 2.5 hrs. At the end of treatment, cells were washed with PBS and fixed in ice-cold methanol for 3 min. Localizations of TLR4, P-p65, and occludin were analyzed as follows. Briefly, the cells were treated with rabbit polyclonal anti-TLR4 or P-p65 antibodies (Cell Signaling Technology), or mouse monoclonal anti-occludin antibody (Zymed Laboratories, Milan). For secondary detection, the cells were incubated with FITC-conjugated goat anti-rabbit IgG for TLR4, TRITC-conjugated goat anti-mouse IgG (Jackson Immunoresearch, Milan) for occludin or TRITC-conjugated goat anti-rabbit IgG for P-p65. For P-p65 analysis, the cell nuclei were stained with 300 nM DAPI, added directly to the mounting medium. Stained monolayers were mounted on glass slides by using Prolong Gold antifade Reagent (Molecular Probes, Invitrogen, Milan) and analyzed under a confocal microscope (LSM 700, Zeiss, Jena, Germany).

### Measurement of inflammatory cytokine production by intestinal cells

Caco-2/TC7 cells differentiated on Transwell filters (1×10^6^ cells/filter), were untreated (control) or apically treated with 1 mL of medium containing ETEC (5×10^6^ CFU/mL), *L. amylovorus* (5×10^7^ CFU/mL) or *L. amylovorus* supernatant (equivalent to 5×10^7^ CFU/mL), either alone or simultaneously with ETEC, for 4 hrs. At the end of bacterial incubation time, antibiotics (10^5^ U/L penicillin and 100 mg/L streptomycin) were added to the apical media for 20 hrs. IL-8 and IL-1β levels were analyzed in basolateral media by a cytometric bead array inflammatory kit (Becton Dickinson Biosciences, Milan), according to the manufacturer's specifications. Briefly, microbeads with distinct fluorescence intensities, coated with capture antibodies specific for each cytokine, were incubated with supernatant samples (50 µL) and PE-conjugated detection antibodies for 2 hrs. The samples were then washed, resuspended in 300 µL wash buffer, and analyzed by flow cytometry using FCAP array software (BD Biosciences).

### Statistical analysis

The significance of the differences was evaluated by one-way ANOVA followed by Fisher's test. Significance was set at P values <0.05. All statistical analyses were performed with “Statistica” software program (version 4.5; StatSoftInc, Tulsa, OK).

## Results

### Inhibition of ETEC induced TLR4 signaling pathways by L. amylovorus in Caco-2/TC7 cells and pig intestinal explants

**Figure 1:**
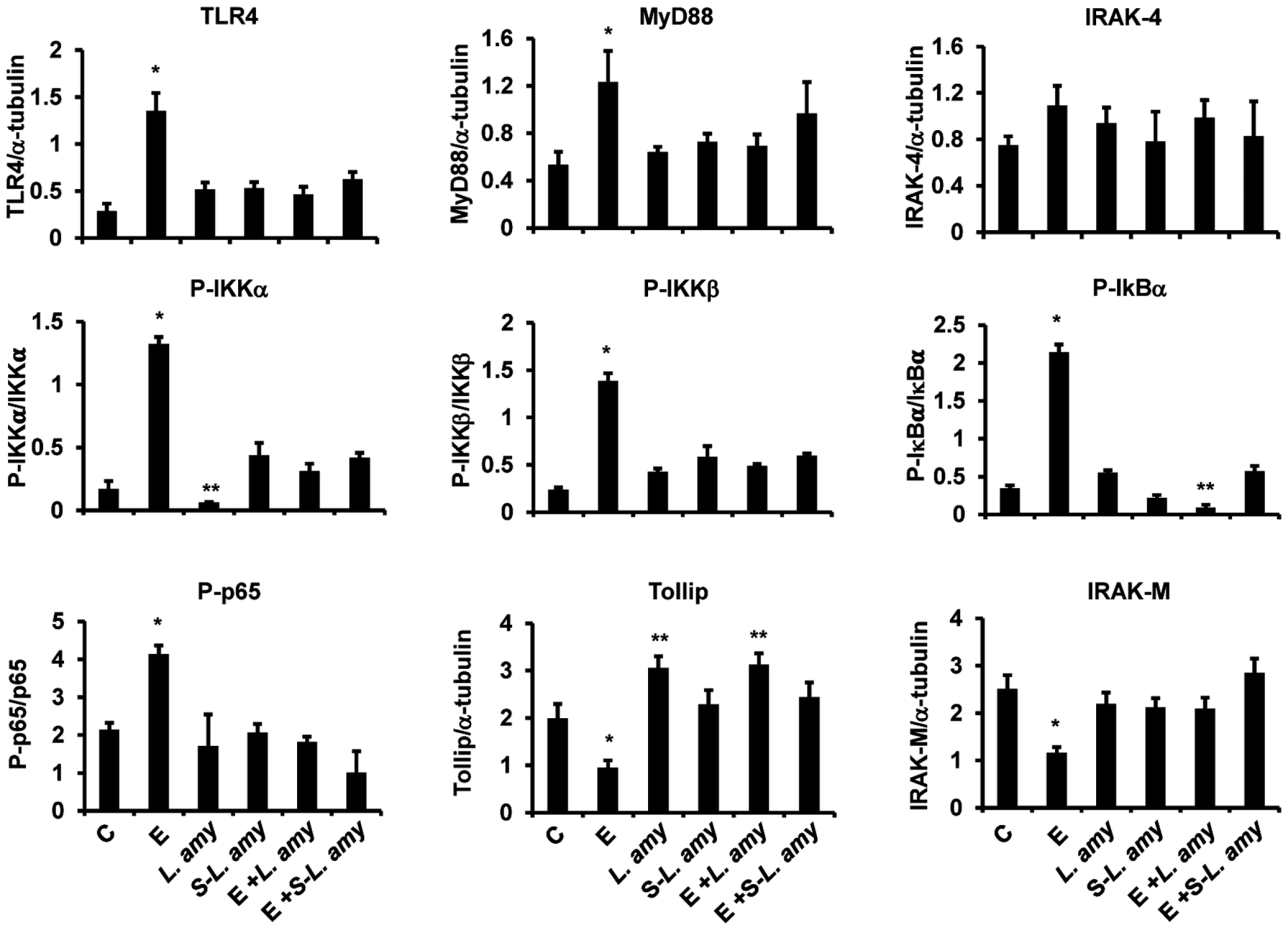
Inhibition of ETEC induced TLR-4 signaling pathways by *L. amylovorus* in Caco-2/TC7 cells. The cells were untreated (control, C), infected with ETEC (E), or treated with *L. amylovorus* (*L. amy*) or its supernatant (S-*L. amy*), either alone or simultaneously with ETEC. The figure shows the densitometric values of proteins involved in TLR4 signaling, analyzed by Western blot. The relative expression levels of TLR4, MyD88, IRAK-4, Tollip and IRAK-M were normalized to α-tubulin, whereas the phosphorylated IKKα, IKKβ, IκBα and p65 were normalized to their corresponding unphosphorylated forms. Values represent means ± SD of three independent experiments, carried out in triplicate. *P<0.01 compared with all. **P<0.05 compared with C.

**Figure 2:**
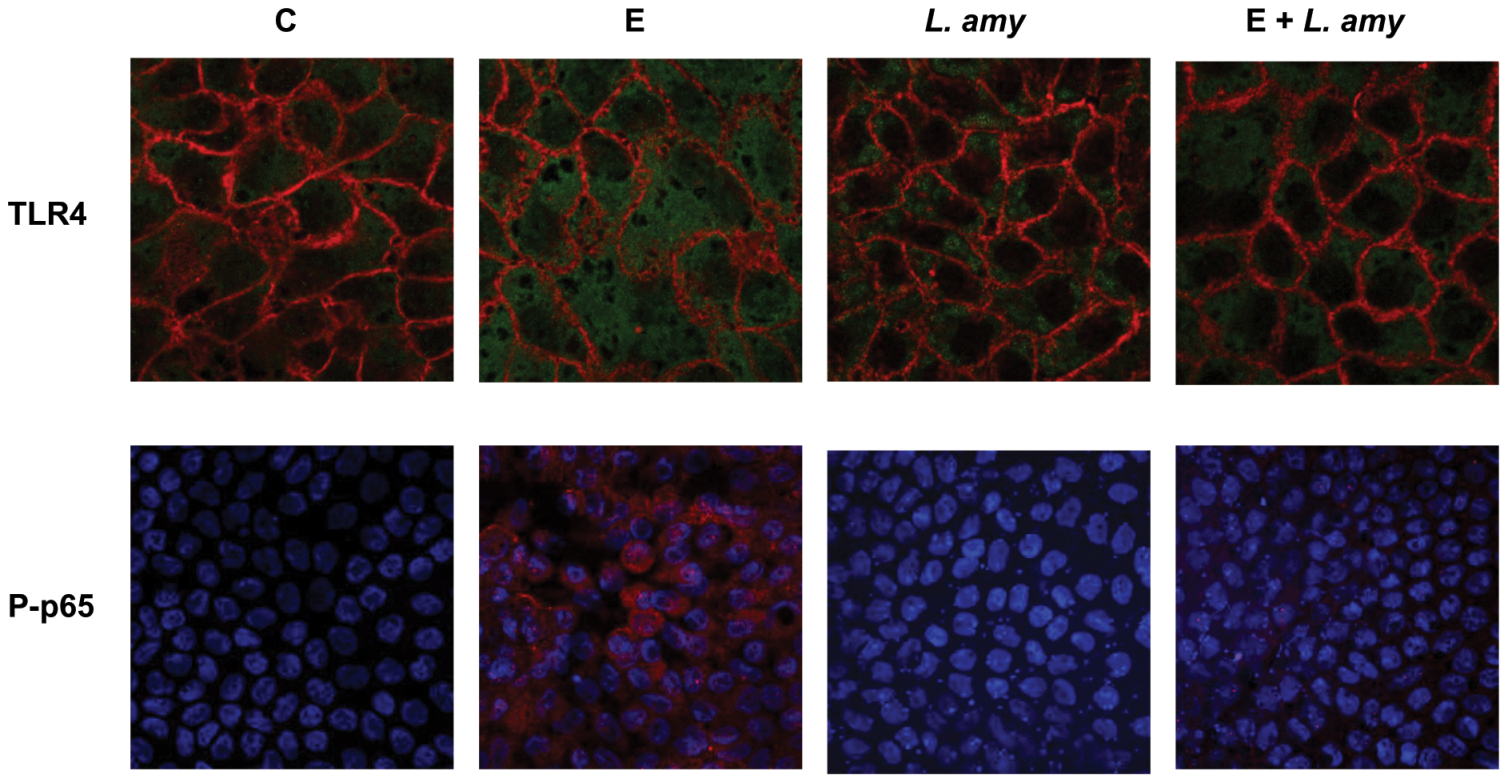
Inhibition of ETEC induced TLR-4 up-regulation and P-p65 translocation into the nucleus by *L. amylovorus.* Caco-2/TC7 cells were untreated (control, C), infected with ETEC (E), or treated with *L. amylovorus* (*L. amy*), either alone or simultaneously with ETEC, and analyzed by immunofluorescence. For TLR4 analysis, cells were stained in green for TLR4 and in red for occludin. For P-p65 analysis, cells were stained in red for P-p65, while nuclei were stained in blue. Each figure is representative of three independent assays (63× and 40× magnification for TLR4 and P-p65, respectively).

**Figure 3:**
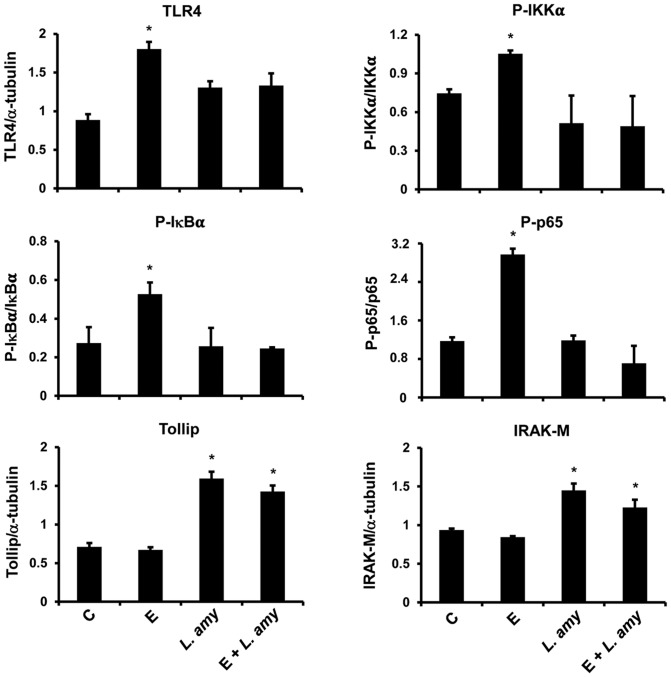
Inhibition of ETEC induced TLR-4 signaling pathways by *L. amylovorus* in intestinal explants. Pig jejunal explants were untreated (control, C), infected with ETEC (E), or treated with *L. amylovorus* (*L. amy*), either alone or simultaneously with ETEC. The figure shows the densitometric values of proteins involved in TLR4 signaling, analyzed by Western blot. The relative expression levels of TLR4, Tollip and IRAK-M were normalized to α-tubulin, whereas the phosphorylated IKKα, IκBα and p65 were normalized to their corresponding unphosphorylated forms. Values represent means ± SD of two independent experiments of five animals each.*P<0.01 compared with all.

Infection of Caco-2/TC7 cells with ETEC resulted in a stimulation of TLR4 inflammatory cascade. ETEC induced a significant increase in the levels of TLR4 and MyD88, and caused a strong phosphorylation of the IKK family proteins IKKα and IKKβ, IκBα and NF-κB subunit, p65 (Figure 1). The immunofluorescence analysis of TLR4 in Caco-2/TC7 cells confirmed the increase of the receptor caused by ETEC, that was abolished by co-treatment with *L. amylovorus* (Figure 2). In addition, the negative regulators Tollip and IRAK-M were reduced by ETEC, as compared with untreated cells. All these inflammatory responses to ETEC were inhibited by *L. amylovorus* and its cell free supernatant. Of relevance, *L. amylovorus* when added simultaneously with ETEC, induced an up-regulation of Tollip, as compared with control cells. Treatment of uninfected cells with *L. amylovorus* did not activate the TLR4 signaling, and induced an increased level of Tollip, as compared with control cells (Figure 1). Similar results were found in pig intestinal explants (Figure 3), where ETEC induced a higher level of TLR4, P-IKKα, P-IκBα, and P-p65, while *L. amylovorus* completely abolished all these alterations and upregulated Tollip and IRAK-M expression when co-treated with ETEC. In addition, *L. amylovorus* did not activate the TLR4 cascade and increased the levels of the two negative regulators when added alone to the tissue culture.

### *L. amylovorus* abolishes P-p65 translocation into the nucleus induced by ETEC

Since phosphorylation of p65 is necessary for its translocation into the nucleus to activate transcription of inflammatory cytokines, we verified whether this final step of TLR4 cascade was inhibited by *L. amylovorus.* The immunofluorescence experiments showed nuclear localization of P-p65 upon ETEC stimulation that was not present in cells simultaneously treated with ETEC and *L. amylovorus* (Figure 2). The P-p65 translocation into the nucleus did not occur after the addition of *L. amylovorus* alone to the cells.

### *L. amylovorus* inhibits the ETEC induced up-regulation of extracellular Hsp72 and Hsp90

**Figure 4:**
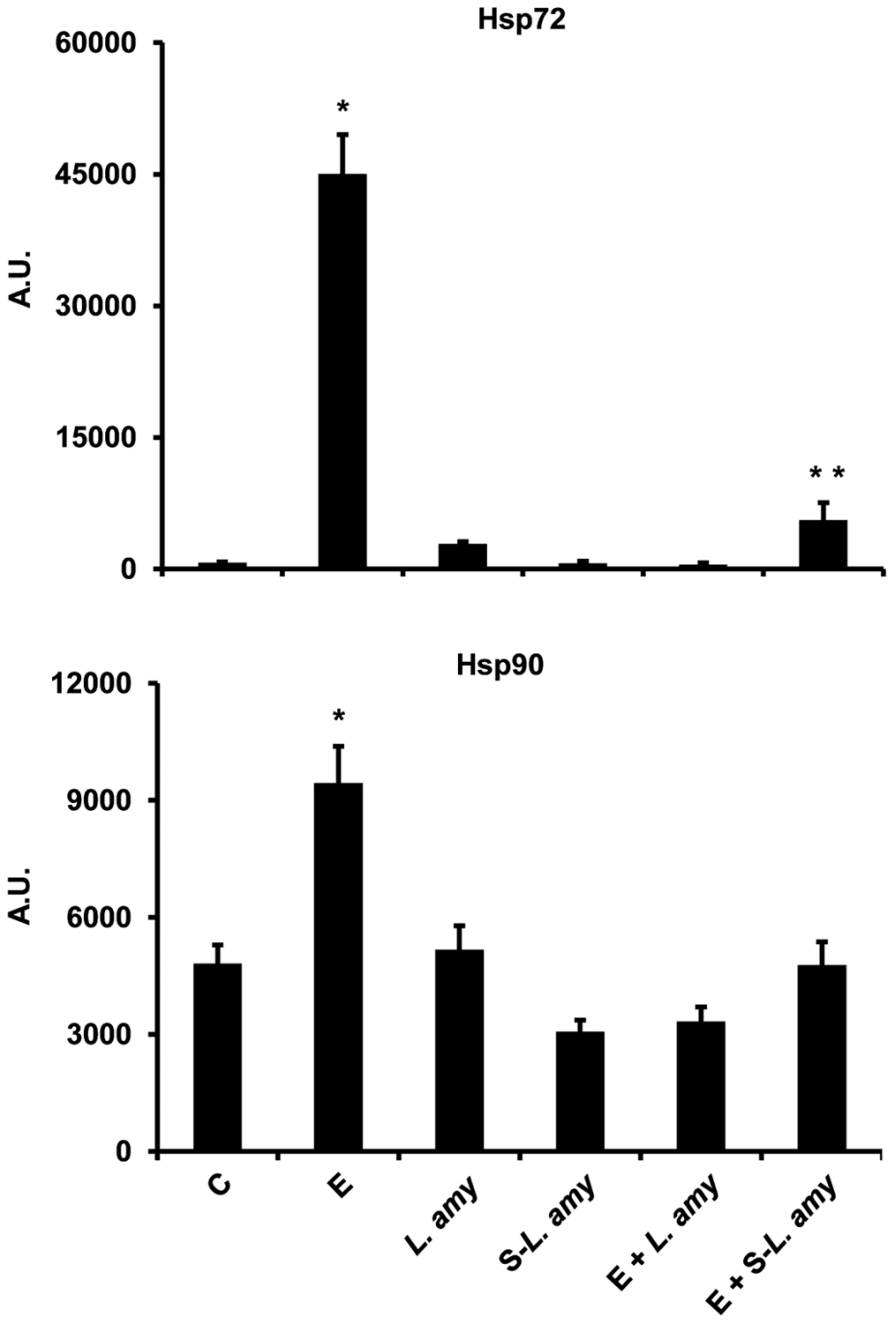
*L. amylovorus* inhibits the ETEC induced up-regulation of extracellular Hsp72 and Hsp90. Caco-2/TC7 cells were untreated (control, C), infected with ETEC (E), or treated with *L. amylovorus* (*L. amy*) or its supernatant (S-*L. amy*), either alone or simultaneously with ETEC. The Hsp72 and Hsp90 levels were analyzed by Western blot and expressed in arbitrary units (A.U). Values represent means ± SD of three independent assays, carried out in triplicate. *P<0.001 compared with all. **P<0.05 compared with C.

Since extracellular Hsps can activate the TLR4 signaling, we further investigated whether these proteins were regulated by ETEC and *L. amylovorus*. The infection of Caco-2/TC7 cells with ETEC caused an increase in Hsp72 and Hsp90 levels, that was inhibited by co-treatment of the cells with ETEC and *L. amylovorus* (Figure 4). Similar effects were obtained when the cells were treated with ETEC and *L. amylovorus* supernatant. No change in the levels of Hsp72 and Hsp90 was induced by treatment with *L. amylovorus* alone.

### *L. amylovorus* abolishes the ETEC induced increase of pro-inflammatory cytokine production

**Figure 5:**
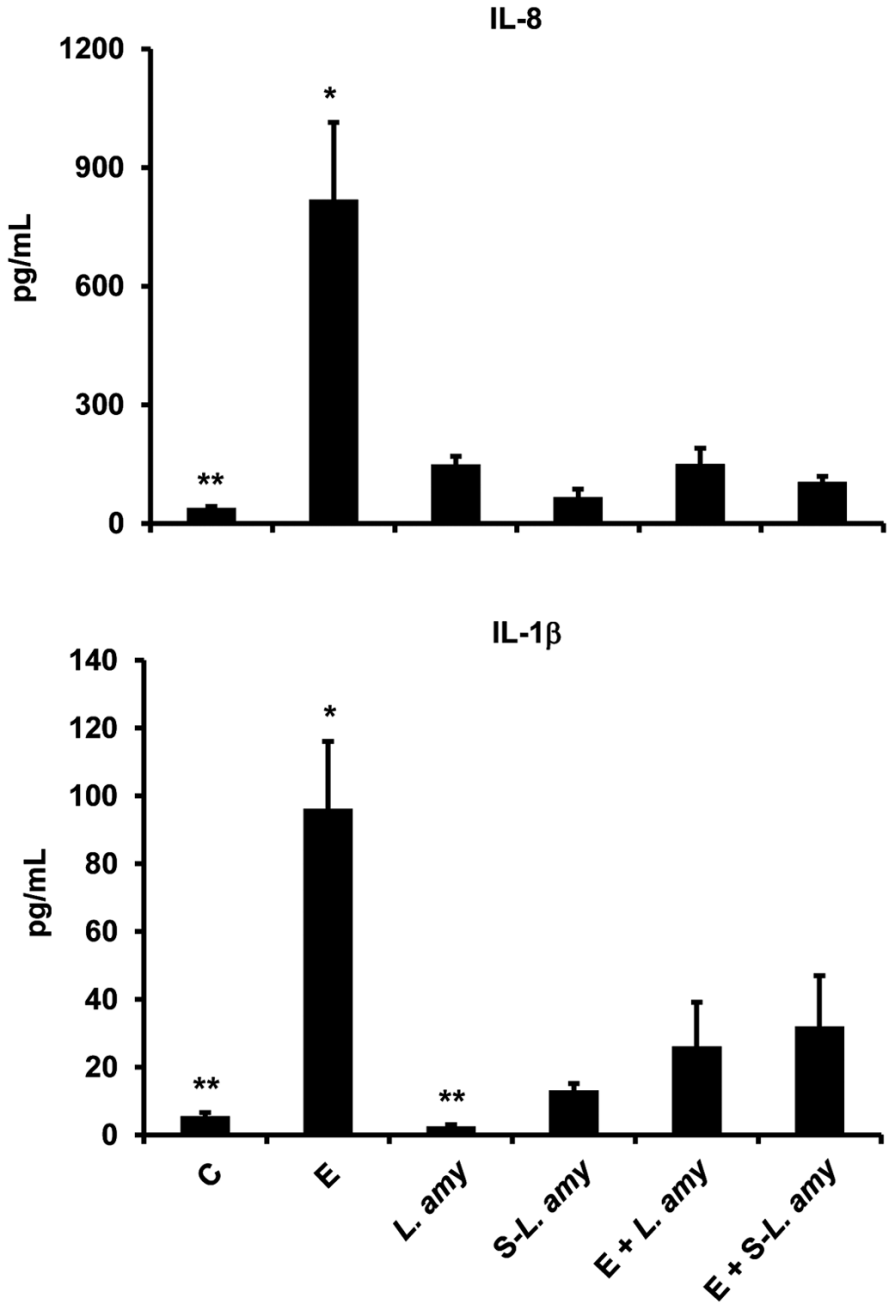
*L. amylovorus* abolishes the ETEC induced increase of pro-inflammatory cytokine production. Caco-2/TC7 cells were untreated (control, C), infected with ETEC (E), or treated with *L. amylovorus* (*L. amy*) or its supernatant (S-*L. amy*), either alone or simultaneously with ETEC. IL-8 and IL-1β levels were analyzed in basolateral media by a cytometric bead array inflammatory kit. Values represent means ± SD of three independent experiments, carried out in triplicate. *P<0.001 compared with all. **P<0.05 compared with C.

A strong secretion of IL-8 and IL-1β was induced by ETEC in Caco-2/TC7 cells, compared with control cells (Figure 5). *L. amylovorus* and its cell free supernatant when added simultaneously with ETEC, were able to inhibit the up-regulation of both these cytokines. The level of IL-8 and IL-1β was not increased by treatment of *L. amylovorus* or its cell free supernatant alone.

### The regulation of TLR4 signaling is TLR2 dependent

**Figure 6:**
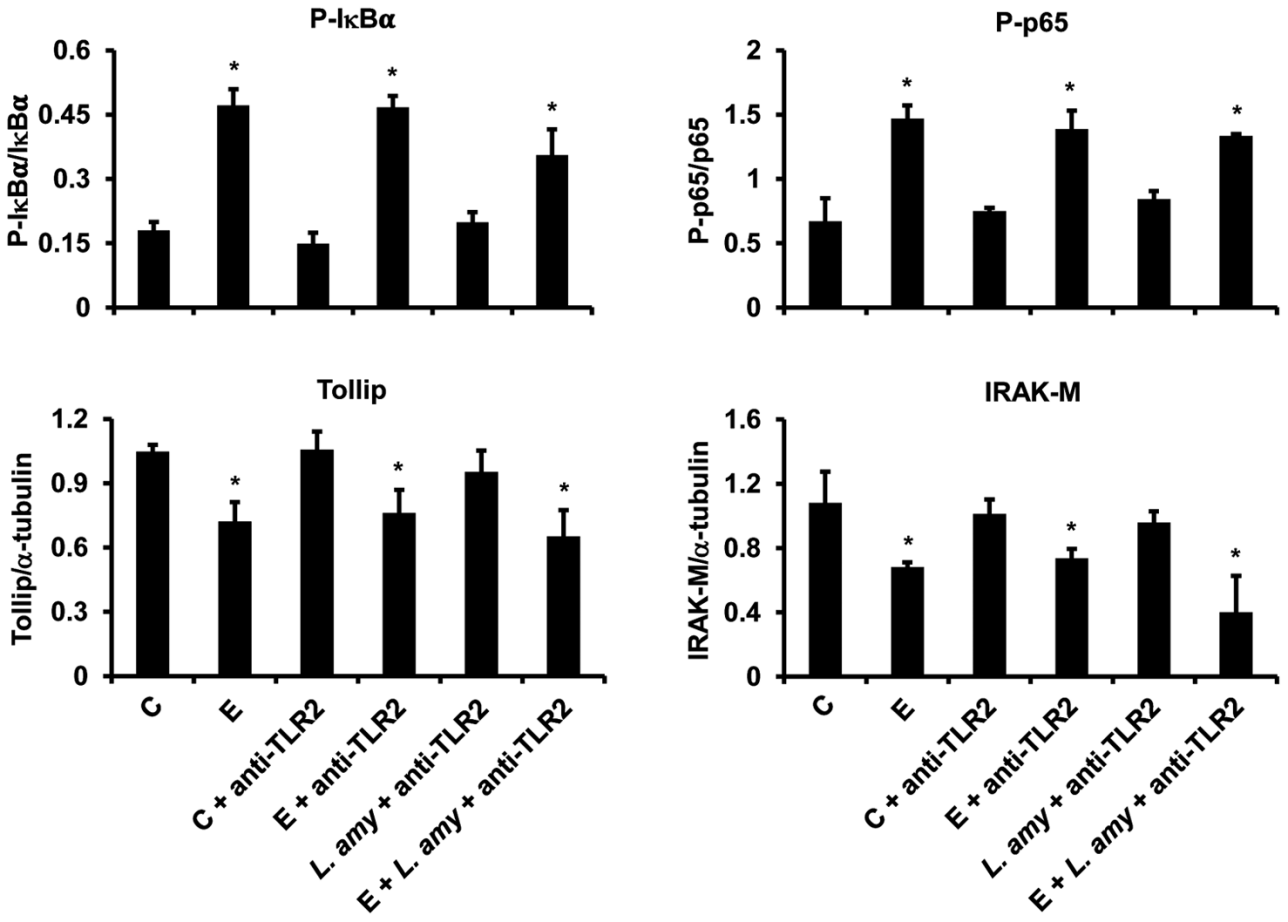
The regulation of TLR4 signaling is TLR2 dependent. Caco-2/TC7 cells were pretreated with anti-TLR2 antibodies and then treated with *L. amylovorus (L. amy)*, either alone or simultaneously with ETEC. Control (C) and ETEC (E) infected cells were not pretreated with anti-TLR2 antibodies. The figure shows the densitometric values of Western blots. The relative expression levels of Tollip and IRAK-M were normalized to α-tubulin, whereas the phosphorylated IκBα and p65 were normalized to their corresponding unphosphorylated forms. Values represent means ± SD of three independent assays, carried out in triplicate. *P<0.05 compared with all.

In order to evaluate whether TLR2 could play a role in *L. amylovorus* anti-inflammatory activity, we performed immunoneutralization experiments using anti-TLR2 antibodies. The neutralization of TLR2 resulted in a lack of protection by *L. amylovorus*, since the levels of P-IκBα and P-p65 were up-regulated, while the levels of Tollip and IRAK-M decreased in cells co-treated with ETEC, *L. amylovorus* and anti-TLR2, as compared with control cells, reaching similar levels to those of ETEC infected cells (Figure 6). The addition of anti-TLR2 antibody to ETEC infected cells did not induce any change in the levels of P-IκBα, P-p65, Tollip and IRAK-M, as compared with ETEC infected-anti-TLR2 untreated cells. The levels of these proteins were not modified by the addition of anti-TLR2 antibody to control and *L. amylovorus* treated cells, as compared with control cells.

## Discussion

In a previous study we showed that *L. amylovorus* DSM 16698^T^ protects the intestinal cells against the inflammatory status and mucosal injury triggered by ETEC K88 infection in intestinal cells through repression of pro-inflammatory cytokine production, such as IL-1β and IL-8. Of relevance, IL-8 was responsible of the ETEC induced membrane barrier damages of the cells [29]. The results of the present study indicate a mechanism through which the protective activity of *L. amylovorus* DSM 16698^T^ is exerted, since we found that *L. amylovorus* blocks the ETEC induced increase in IL-8 and IL-1β by inhibiting the various steps of TLR4 signaling and modulating the cascade negative regulators. These findings are consistent with the notion that TLR4 down-regulation is important for the resolution of inflammation and repair of membrane damage, as established by previous studies showing that TLR4 knockout mice had reduced inflammation in response to pathogen infection [39], and that TLR4 antagonist inhibited pro-inflammatory cytokine production and mucosal damages in dextran sodium sulfate-induced colitis mice and in spontaneous chronic colitis model [40]. In addition, downregulation of TLR4 expression in pigs challenged with LPS was associated with a decrease in pro-inflammatory cytokine levels and improvement of intestinal barrier integrity [41]. Our results contribute to the understanding of the mechanisms through which probiotics may inhibit the intestinal injury caused by pathogenic infection. In fact, while the role of probiotics in the maintenance of membrane barrier function is supported by several studies [29], [42], only recently their protective activity through modulation of TLR signaling has been addressed, but the results are sometime controversial (26–28).

We show that the results obtained in intestinal cells are confirmed in porcine explants treated with ETEC K88 and *L. amylovorus* DSM 16698^T^, supporting the ability of this lactobacillus to prevent or reduce the inflammatory response to ETEC infection in piglets through regulation of the TLR4 signaling. Notably, a previous study showed that *L. amylovorus* DSM 16698^T^ (formerly called *L. sobrius*) was effective in reducing ETEC infection and in improving weight gain of piglets [43].

Pathogen binding to TLR4 triggers aggregation of TLR4 with its co-adaptor MyD88, which initiates the inflammatory cascade leading to the production of pro-inflammatory cytokines such as TNF-α, IL-1β, IL-6 and IL-8 [19], [44]. Our results show an elevated level of TLR4 and MyD88 after ETEC infection, in agreement with the findings of an increased expression of TLR4 in Caco-2 cells after *E. coli* K4 infection [45]. Treatment of ETEC infected cells with *L. amylovorus* or its cell free supernatant inhibits the increase in both TLR4 and MyD88, indicating that *L. amylovorus* is able to block the first steps of the TLR4 inflammatory signaling. An ability to downregulate the expression of TLR4 has been recognized in other lactobacillus strains. For instance, *L. paracasei* inhibited the increase in TLR4 caused by *Salmonella* infection [26]. Treatment with *L. reuteri* induced a down-regulation of TLR4 associated with a reduction of inflammation in experimental enterocolitis [46]. By contrast, *L. rhamnosus* and *L. plantarum* did not modify the TLR4 expression in cells infected with *Salmonella* [28]. All together, these data indicate that the regulation of TLR4 expression may depend on the bacterial strain, and that TLR4 is a target of *L. amylovorus* anti-inflammatory activity. In uninfected cells, *L. amylovorus* does not modify the expression of TLR4 and MyD88, and this result was expected since TLR4 is the essential receptor for Gram-negative bacteria. Accordingly, previous studies showed no influence on TLR4 gene expression by lactic acid bacteria in Caco-2 cells [45], [47]. Of relevance, our data underline that the immunomodulatory activity of *L. amylovorus* may change with the presence or absence of pathogens, suggesting that this property, likely extendable to other lactobacillus strains, should be considered for attributing anti-inflammatory properties to lactobacilli.

Another way by which *L. amylovorus* may counteract the ETEC induced inflammation is the inhibition of the extracellular secretion of Hsp72 and Hsp90. These proteins induce inflammation and are critical for the regulation of TLR4 complex formation and function [13]–[17], [48]. In fact, the increase in IL-8 secretion induced by extracellular Hsp90 was suppressed by a dominant-negative form of TLR4 in vascular smooth muscle cells [16]. Furthermore, the induction of IL-8 production by extracellular Hsp72 in leukemic HL-60 cells was dependent upon activation of TLR4 and NF-κB [14]. In the present study we found strong increases in the secretion of both Hsp72 and Hsp90 after ETEC infection, which are associated with TLR4 up-regulation. These findings are consistent with previous studies showing extracellular release of Hsp72 in virally infected airway epithelial cells via activation of TLR4 [14]. Although further experiments are necessary to verify whether the reduction in Hsp72 and Hsp90 is associated with inhibition of Hsp72- and Hsp90-TLR4 binding, our results reasonably indicate that the down-regulation of the extracellular Hsp72 and Hsp90 by *L. amylovorus* contributes to the inhibition of TLR4 inflammatory signals. In addition, we demonstrate that the factor/factors secreted by *L. amyovorus* are able to inhibit the Hsp72 and Hsp90 release, as shown by the decreased levels of Hsp72 and Hsp90 in cells treated with *L. amylovorus* supernatant. To our best knowledge, this is the first study showing the ability of a lactobacillus strain and of its secreted products to regulate the expression of extracellular Hsps that are involved in TLR4 mediated inflammatory response to a pathogen.

The classical signaling pathway of NF-κB activation triggered by pathogens includes activation of the IKK complex followed by phosphorylation and degradation of IκB proteins, that allow phosphorylation of p65 subunit and NF-κB translocation into the nucleus to activate the transcription of inflammatory genes [19]–[22]. In the present study, ETEC activates all these steps leading to higher levels of IL-1β and IL-8. On the other hand, *L. amylovorus* is able to block the phosphorylation of the IKK complex, IκBα and p65, and consequently to inhibit the enhanced production of IL-1β and IL-8. Interestingly, similar inhibitory effects are triggered by *L. amylovorus* supernatant. These results together with those on Hsp72 and Hsp90, indicate that the released soluble factors from *L. amylovorus* possess anti-inflammatory activity and may act throughout the TLR4 cascade, by inhibiting either the microbial receptor with the involvement of the extracellular Hsp72 and Hsp90, or the steps downstream TLR4, up to the inhibition of the pro-inflammatory cytokine production. Recently, an ability of *L. paracasei* and *B. breve* supernatants to modulate TLR signaling and reduce pro-inflammatory cytokine secretion in dendritic cells challenged with *S. typhi* was shown [26], [49]. However, contrary to our results, these studies also found an up-regulation of TLR4 or TLR9, suggesting different anti-inflammatory properties of probiotic secreted products.

The inhibition of the NF-κB signaling may be achieved through the activity of negative regulators such as Tollip and IRAK-M [50], [51]. The important role played by these regulators to control the TLR induced inflammatory responses was shown by several studies. Tollip-deficient mice mounted a decreased immune response to LPS stimulation [52]. The expression of Tollip was up-regulated in intestinal cells hyporesponsive to TLR activation, and overexpression of Tollip resulted in decreased pro-inflammatory response [53]. In addition, IRAK-M-deficient cells stimulated by TLR ligands or bacteria had increased NF-κB activation and pro-inflammatory cytokines production [50], [54]. Less clear is whether the negative regulators are the targets of pathogen infection. For instance, IRAK-M was up-regulated in LPS treated macrophages or in lung epithelial cells in response to *S. pneumonia* [50], [55], while ETEC infection did not modify IRAK-M and Tollip expression in bovine epithelial cells, and *S. typhi* caused a decrease in Tollip level in dendritic cells [26], [56]. Our results show a decrease in Tollip and IRAK-M levels triggered by ETEC infection in Caco-2/TC7 cells, whereas these negative regulators are unaffected by ETEC in intestinal explants. Despite these different effects of ETEC, our findings in both intestinal cells and pig explants clearly indicate that the repression of the NF-κB signaling involves modulation of Tollip and IRAK-M. In addition, we report that *L. amylovorus* and its supernatant up-regulate Tollip either in the presence or absence of ETEC. These findings are in line with those of previous studies showing an enhanced Tollip gene expression in dendritic cells upon stimulation with *S. typhi* and *B. breve* [26], and in bovine epithelial intestinal cells treated with *L. casei* alone or simultaneously with ETEC [56]. However, Tollip gene expression was unaffected or increased by the addition of several strains of lactobacilli in uninfected porcine intestinal epithelial cells [27], [57], suggesting a strain-specific effect on Tollip expression. Our study by investigating the regulation of Tollip protein expression by *L. amylovorus* in both uninfected and pathogen infected cells, provides advanced knowledge of Tollip modulation by lactobacilli. The inhibition of TLR4 signaling is triggered as well via modulation of IRAK-M, since *L. amylovorus* is able to counteract its decrease induced by ETEC in Caco-2/TC7 cells or even to up-regulate its level in pig explants. There is some evidence that lactobacilli can modulate the expression of IRAK-M, however the related studies were conducted in uninfected cells. In fact, Biswas *et al.* showed that IRAK-M activity was restored in germfree mice by colonization with *L. plantarum* [58]. Villena *et al.* reported that the immunomodulatory effect of *L. jensenii* in porcine antigen-presenting cells was dependent on the increased expression of three negative regulators of TLRs, including IRAK-M [59]. Thus, our results show a novel ability of lactobacilli, specifically of *L. amylovorus* DSM 16698^T^, to counteract the pathogen induced activation of TLR4 signaling by controlling the IRAK-M protein expression.

The intestinal epithelial cells are exposed to a myriad of commensal and pathogenic bacteria, and we cannot exclude that other cascades than TLR4 signaling may be stimulated by ETEC infection, all leading to NF-κB activation, and possibly *L. amylovorus* dampens all these signaling. Further studies are necessary to elucidate these aspects. We hypothesized that activation of TLR2 by *L. amylovorus* binding may be required for the anti-inflammatory activity. This hypothesis was supported by previous findings that treatment of epithelial cells with anti-TLR2 antibody abolished the *L. plantarum* induced blockade of IL-17 and IL-23 production triggered by *S. pyogenes* infection [60]. In addition, anti-TLR2 antibody blocked the up-regulation of IRAK-M in porcine antigen-presenting cells [59], and activation of TLR2 reduced the mucosal inflammation in mice [61]. Our results show for the first time that the binding of *L. amylovorus* to TLR2 is necessary for the inhibition of TLR4 signaling steps, as well as for the regulation of Tollip and IRAK-M, further supporting the relevance of probiotic mediated TLR2 activation to counteract the inflammatory signaling and restore intestinal immune balance.

In conclusion, the results reported in this manuscript provide advance in the knowledge of the mechanisms of probiotic anti-inflammatory activity by demonstrating that *L. amylovorus* DSM 16698^T^ and its cell free supernatant inhibit the ETEC K88 induced activation of the TLR4 signaling pathway through modulation of the negative regulators Tollip and IRAK-M, as well as down-regulation of the extracellular Hsp72 and Hsp90, which are important for TLR4 functioning, leading to reduced pro-inflammatory cytokine production. In addition, we show that these anti-inflammatory activities are TLR2 dependent. Of relevance, the results obtained in the *in vitro* model of Caco-2/TC7 cells were confirmed in the *ex vivo* model of piglets mucosal explants. This study may provide helpful information for the development of potential therapeutic strategies using *L. amylovorus* to prevent or ameliorate intestinal disorders in piglets and humans. Notably, a recent study indicates that *L. amylovorus* may be considered a probiotic strain for animal as well as for human health [31].
